# Supplementary material for: The cell adhesion molecule TMIGD1 binds to moesin and regulates tubulin acetylation and cell migration
Source: J Biomed Sci. 2021 Sep 9;28:61. doi: 10.1186/s12929-021-00757-z (PMC8427838; doi:10.1186/s12929-021-00757-z)

Supplemental Data

The Cell Adhesion Molecule TMIGD1 Binds to Moesin and Regulates Tubulin Acetylation and Cell Migration

Nader Rahimi[1], Rachel X-Y Ho[1], Kevin Brown Chandler[2], Kyle Oliver Corcino De La Cena[1], Razie Amraei[1], Ashley J Mitchel[1], Nels Engblom[1], Catherine E Costello[2]

[1]Department of Pathology, School of Medicine, Boston University Medical Campus, Boston, MA 02118.
[2]Center for Biomedical Mass Spectrometry, Boston University School of Medicine, Boston, MA 02118.

Key Words: TMIGD1, Moesin, Ezrin, tubulin acetylation, mitotic spindle, microtubular, tumor suppressor

Running Title: TMIGD1/ERM Axis Regulates tubulin acetylation and cell migration
Conflict of Interest: Authors declare no conflict of interest.
Authors' Contributions: NR, CEC and RX-YO were involved in writing and editing of the manuscript. NR, KBC, KCSDC, RA, AM and NE all were involved in the design and performing the experiments.
Corresponding Authors: Nader Rahimi, nrahmi@bu.edu and Catherine E Costello, cecmsms@bu.edu.
Funding information: This work was supported in part through grants from CTSI grant (UL1TR001430) and Malory Fund, Department of Pathology, Boston University (NR), P41 GM104603 (CEC) and R24 GM134210 (CEC).

S. Figure 1: Amino acid sequence homology of human moesin and ezrin. Alignment of human moesin (MSN) and ezrin (EZN) is shown. The FERM domain is shown in a box. Hydrophobic and negatively amino acids are highlighted in light blue and light brown colors, respectively.

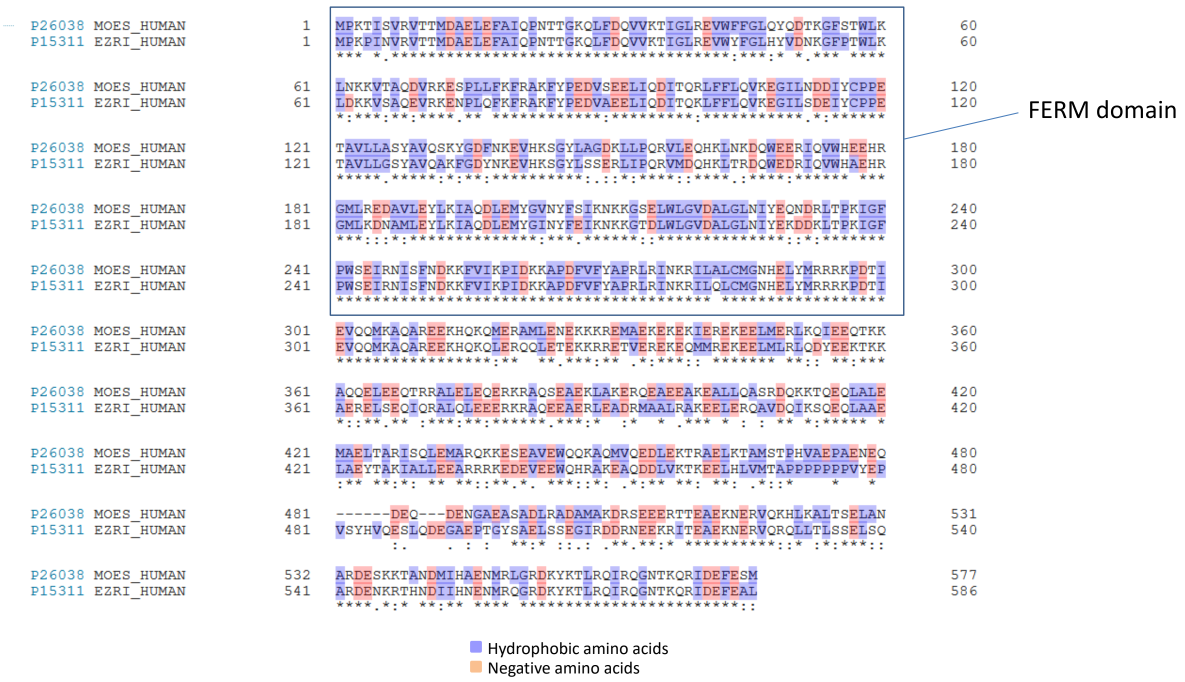

S. Figure 2. qPCR analysis of TMIGD1 KO mouse: qPCR analysis of wild-type and TMIGD1 is shown.

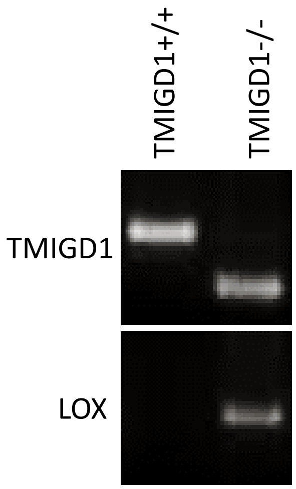

S. Figure 3. Ectopic expression of Moesin and TMIGD1 in HEK-293 cell. Whole cell lysates derived from HEK-293 cells expressing TMIGD1 or moesin subjected to western blot analysis.

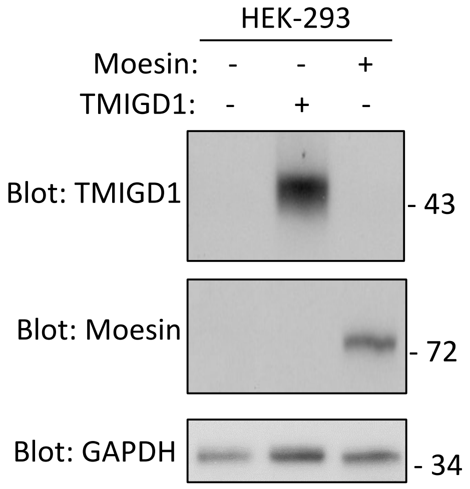

S. Figure 4: **TMIGD1, Moesin and Ezrin mRNA levels in human normal kidney verses kidney cancer types**. (**A-C**) TMIGD1, Moesin and Ezrin mRNA levels obtained from the TCGA data set via Fire Brows (http://firebrowse.org/). Kidney cancer types; kidney clear cell renal cell carcinoma (KIRC), kidney papillary renal cell carcinoma (KIRP), chromophobe renal cell carcinoma (KICH) and kidney pan cancer analysis (KIPAN). The horizontal small black bars within each box correspond to median expression of given proteins.

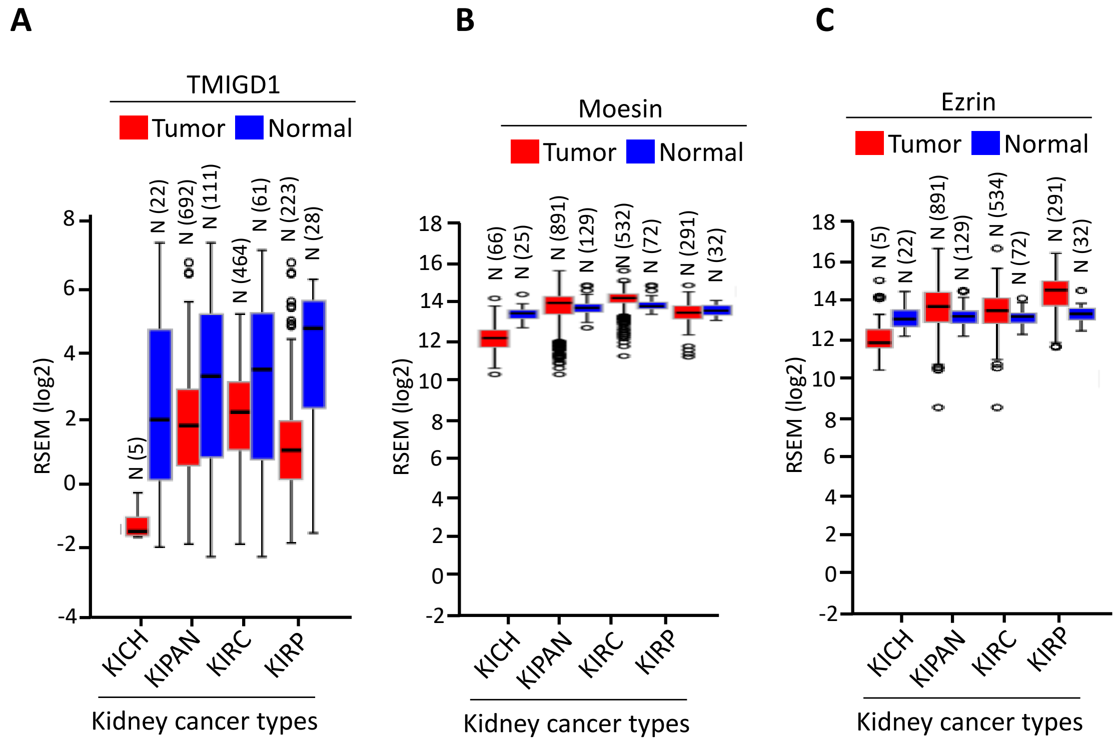

Supplement: Supplementary file 1 — Additional file 1: Figure S1. Amino acid sequence homology of human moesin and ezrin. Alignment of human moesin (MSN) and ezrin (EZN) is shown. The FERM domain is shown in a box. Hydrophobic and negatively amino acids are highlighted in light blue and light brown colors, respectively. Figure S2. qPCR analysis of TMIGD1 KO mouse: qPCR analysis of wild-type and TMIGD1 is shown. Figure S3. Ectopic expression of Moesin and TMIGD1 in HEK-293 cell. Whole cell lysates derived from HEK-293 cells expressing TMIGD1 or moesin subjected to western blot analysis. Figure S4. TMIGD1, Moesin and Ezrin mRNA levels in human normal kidney verses kidney cancer types. A–C TMIGD1, Moesin and Ezrin mRNA levels obtained from the TCGA data set via Fire Brows (http://firebrowse.org/). Kidney cancer types; kidney clear cell renal cell carcinoma (KIRC), kidney papillary renal cell carcinoma (KIRP), chromophobe renal cell carcinoma (KICH) and kidney pan cancer analysis (KIPAN). The horizontal small black bars within each box correspond to median expression of given proteins. [file 12929_2021_757_MOESM1_ESM.pdf]
